# Orally Administered Crocin Protects Against Cerebral Ischemia/Reperfusion Injury Through the Metabolic Transformation of Crocetin by Gut Microbiota

**DOI:** 10.3389/fphar.2019.00440

**Published:** 2019-04-30

**Authors:** Yue Zhang, Jianliang Geng, Yu Hong, Li Jiao, Shuning Li, Runbin Sun, Yuan Xie, Caixia Yan, Jiye Aa, Guangji Wang

**Affiliations:** Key Laboratory of Drug Metabolism and Pharmacokinetics, State Key Laboratory of Natural Medicines, China Pharmaceutical University, Nanjing, China

**Keywords:** crocin, cerebral ischemia-reperfusion, drug metabolism, gut microbiota, metabolomics, pharmacokinetics

## Abstract

Our pilot study suggested that orally administered crocin was hardly absorbed into circulatory system, but it was effective against cerebral ischemic/reperfusion (I/R) injury. The pharmacologically active component and targeting site of crocin remain elusive. In this study, the cerebral-protective effect of crocin was evaluated on a rat transient middle cerebral artery occlusion (MCAO) model. Our data showed that oral administration of crocin had better effectiveness in cerebral protection than an intravenous injection. Neither crocin nor its metabolite crocetin were determined in the brain of cerebral I/R rats, indicating a target site of periphery. Abundant crocetin was detected in plasma after oral administration instead of intravenous injection of crocin. Meanwhile, orally administered crocetin showed similar cerebral protection to that of crocin, but this exciting effect was not clearly observed by intravenous administration of crocetin, indicating the importance of crocetin in gut. Moreover, orally administered crocin showed less cerebral-protective effect in pseudo germ-free (pGF) MCAO rats. *In vitro* and *in vivo* experiments confirmed that crocin could be deglycosylated to crocetin in gut content of normal rats, rather than that of pGF rats, indicating that gut microbiota facilitated the transformation of crocin into crocetin, which played a key role in the activation of the pharmacological effect. Metabolomic study revealed that microbial-host co-metabolic molecules were significantly perturbed after oral administration of crocin, indicating a regulation on intestinal ecosystem. It was further suggested that gut microbiota may be the potential target of the cerebral-protective effect of crocin.

## Introduction

Stroke is a highly prevalent disease and a major cause of death and disability (Rothwell et al., 2011). To date, only one licensed pharmacological agent, rt-PA, has been launched into market to treat acute ischemic stroke patients within 3 h after the ischemic onset (Durukan and Tatlisumak, 2007; Wardlaw et al., 2012; Catanese et al., 2017). There is no alternative oral drug, especially for the patients who have already had ischemic vascular events (Nenci and Goracci, 2000).

Natural medicines serve as excellent sources for the development of modern drugs (Newman, 2008; Li and Vederas, 2009; Li et al., 2018; Liu et al., 2018). Saffron has been validated by long historical use for its cardiac-cerebral vascular benefits (Xi and Qian, 2006; Liu et al., 2016; Rodriguez-Ruiz et al., 2016). The main active ingredients of saffron are crocin (highly hydrophilic) and its aglycone, crocetin (highly lipophilic) (Bathaie and Mousavi, 2010; Amin and Hosseinzadeh, 2012; Milani et al., 2017; Pitsikas and Tarantilis, 2017). Considering that the traditional method of saffron intake is oral administration, the water-soluble component, crocin, is supposed to account for the pharmacological activity of saffron. However, only trace levels of crocin was found in the systemic circulation after oral administration (Asai et al., 2005; Xi et al., 2007; Lautenschlager et al., 2015), while the system exposure of its metabolite, crocetin, is 56- to 81-fold higher than crocin, suggesting that crocetin may be the active component potentially responsible for the pharmacological effects of crocin (Zhang et al., 2017).

It was reported that crocin has protective effects against cerebral ischemic reperfusion injury by suppressing cerebral oxidative stress and increasing the capacity of antioxidant enzymes in the brain (Ochiai et al., 2007; Zheng et al., 2007; Sarshoori et al., 2014; Vakili et al., 2014; Oruc et al., 2016). The obvious limitation is that these studies were merely focused on the cerebral, however, our results indicated that neither crocin nor its metabolite, crocetin, could cross the blood-brain barrier. As far as we know, no pharmacokinetic and pharmacodynamic association studies of crocin have been reported; the exact pharmacologically active ingredient and targeting site remain elusive. Here, we explored the cerebral-protective effects of crocin with a cerebral I/R rat model receiving either oral or intravenous administration. It was found that oral administration of crocin elicited significant cerebral-protective effects, but intravenous administration was less effective. We also provide the potential target of orally administered crocin through a reverse pharmacokinetics approach (Hao et al., 2014).

## Materials and Methods

### Animals

Male specific-pathogen-free Sprague Dawley (SD) rats (240 – 280 g) were purchased from Beijing Vital River Laboratory Animal Technology Co., Ltd. (Beijing, China) and acclimatized in the animal facility for 7 days before experimentation. All rats were housed on autoclaved bedding with a standard laboratory diet and water *ad libitum*, under a 12 h light-dark cycle at 22 ± 2°C. All animal procedures were approved by the Animal Ethics Committee of China Pharmaceutical University (2016-PKPD-05-01).

### Agents and Materials

**FIGURE 1:**
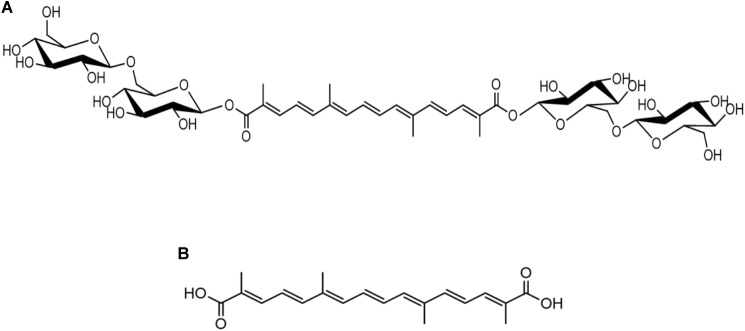
Chemical structures of crocin **(A)** and crocetin **(B)**.

Crocin (PubChem CID: 5281233, purity: 99.33%) and crocetin (PubChem CID: 5281232, purity: 96.61%), Figure 1, were purchased from Chengdu Biopurify Phytochemicals Ltd. (Chengdu, China). The Compound Edaravone Injection was provided by Jiangsu Simcere Pharmaceutical Co., Ltd. (Nanjing, China). 2,3,5-Triphenyltetrazolium chloride (TTC), stable-isotope-labeled IS 1,2-^13^C_2_-myristic acid, ES methyl stearate, methoxyamine hydrochloride, pyridine, hemin, resazurin sodium salt, L-cysteine, 2-ethylbutyric acid, and cholic acid-2,2,4,4-d_4_ were purchased from Sigma-Aldrich (St. Louis, United States). *N*-Methyl-*N*-trimethylsilyltrifluoroacetamide (MSTFA) plus 1% trimethylchlorosilane (TMCS) was purchased from Thermo Scientific (Bellefonte, United States). Streptomycin sulfate and neomycin sulfate were purchased from Nanjing Xinhou Biological Technology Co., Ltd. (Nanjing, China). Yeast extract and tryptone were purchased from Oxoid (Basingstoke, United Kingdom).

### Animal Model and Treatments

#### MCAO Model and Drug Treatments

The rat transient MCAO model was induced as described elsewhere (Geng et al., 2017). Briefly, rats were anesthetized with 10% chloral hydrate (1 mL/300 g, i.p.), and a heating pad was used to maintain the rectal temperature at 37 ± 0.5°C throughout surgery. A monofilament with a silicon-coated tip was inserted into the right external carotid artery and advanced until it obstructed the right middle cerebral artery. The blood circulation was blocked for 2 h and then restored (reperfusion) by removing the monofilament. Sham-operated rats received the same surgical procedure except for the insertion of the monofilament into the external carotid artery. Then, the rats were kept in their home cage with free access to food and water.

The Compound Edaravone Injection (3 mg/kg, i.v.) was used as a positive control and administered at 2 h after the onset of ischemia (Ren et al., 2015; Fujiwara et al., 2016). The sham-operated and model rats were intravenously administered with the same volume of saline. For intravenous (i.v.) administration, crocin (1 mg/kg) was administered at 2 h after the onset of ischemia. For oral (i.g.) administration, crocin (60 mg/kg) was administered once daily for 4 days prior to and on the same day of the MCAO procedure. The doses of crocin were selected according to the pharmacokinetic study (Zhang et al., 2017). Brain tissue samples were collected at 24 h after MCAO for analyses of the infarct volume, biochemical assays and metabolomics studies.

To compare the cerebral and plasma exposure of crocin and crocetin in normal and cerebral I/R rats, two sham-operated groups and two model-operated groups were separately orally and intravenously administered crocin (treated as described above). The plasma and brain tissue samples were collected at 0.25, 0.5, 1, 2, and 4 h after reperfusion, and stored at -70°C until further analysis.

To evaluate the effect of crocetin on cerebral I/R injury, crocetin was treated at equal molar dose to crocin (0.33 mg/kg for intravenous administration and 20 mg/kg for oral administration).

#### Pseudo Germ-Free Rats

Pseudo germ-free rats were established by antibiotic treatment as previously reported (Liu et al., 2012; McCabe et al., 2015; Xiao et al., 2016). Streptomycin sulfate and neomycin sulfate were orally administered at a dosage of 100 mg/kg twice daily for 6 days. The MCAO model was performed on the 7th day, and crocin was orally administered (60 mg/kg) once daily (2 h after the antibiotic treatment) from the 4th day and on the same day of the MCAO model procedure.

### Evaluation of Pharmacological Effects

#### Behavioral Testing

Neurological deficit score was performed 24 h after ischemia by a well-trained observer who was blind to the treatments (Saver et al., 2015). A 5-point scale evolution criterion was performed as follows: 0, equates to no deficit; 1, equates to failure of flexing left forepaw fully; 2, equates to circling counterclockwise; 3, equates to leaning to the affected side; 4, equates to no autonomous activity and unconsciousness. Rats were excluded from the study if no deficit was observed after reperfusion.

#### Measurement of Infarct Volume

Perfused brains were removed and frozen at -20°C for 15 min and then serially sectioned into 6 slices for TTC staining. Slices were incubated in 2% TTC for 30 min and protected from light during the incubation. The normal tissue areas were stained red, and the infarct areas were still in white. The stained sections were scanned by a scanner at 600 dpi. Infarct area was measured using Image-Pro Plus 6.0 software. An edema correction for infarct volume was calculated using the following formula: (ischemic area) = (direct lesion volume) - [(ipsilateral hemisphere) - (contralateral hemisphere)]. The total infarct volume for each rat was calculated by integrating the measured areas and intervals between the sections, and the average infarction volume for each group was statistically analyzed (Singh et al., 2016).

#### Biochemical Assay

Brain tissues of the infarction region (0.02 g) were homogenized (1:10, w:v) in sodium phosphate buffer. The level of MDA and total antioxidant capacity were measured directly from the brain homogenate by using commercially available kits (Beyotime, Shanghai, China, #S0131, #S0116, respectively). The detailed experimental manipulations were performed according to the manufacturer’s instructions. The absorbance was detected using a microplate spectrophotometer (BioTek Instruments, Inc., Winooski, VT, United States). The MDA concentration and the total antioxidant capacity of the brain samples were statistical analyzed after correction with protein concentration.

### Metabolomics Study and Multivariate Statistical Analysis

The metabolomics study of brain tissues was performed on a well-developed GC-MS platform, as previously reported (Jiye et al., 2005; Guo et al., 2016; Geng et al., 2017). Briefly, brain tissues (30 mg) were extracted by 800 μL of 80% methanol (with 5 μg/mL IS). The tissues were homogenized and centrifuged, and 200 μL of supernatant was transferred into the new GC autosampler vials and evaporated to dryness in a rotary evaporator (ThermoFisher Savant SpeedVac Concentrator System, United States). Methoxyamine pyridine solution (10 mg/mL, 30 μL) was added to the dried residue and allowed to stand for 16 h at room temperature. MSTFA plus 1% TMCS, 30 μL, was added and incubated for 1 h at room temperature. Finally, 30 μL of methyl stearate-heptane solution (ES, 30 μg/mL) was added and vortexed for 30 s to mix well. GC-MS analysis was profiled on a GCMS-QP2010 Ultra (Shimadzu, Japan) system. The system includes an automatic sample injector and an Rtx-5 MS capillary column, and helium was used as the carrier gas. The injection parameters were set as described elsewhere (Guo et al., 2016). Samples were assigned in random order and 0.5 μL of the sample aliquot was injected into the GCMS-QP2010 Ultra for analysis.

The metabolites were identified by comparing the MS spectra of each peak in the experimental samples with the MS spectra of the compound in standard libraries, such as the National Institute of Standards and Technology (NIST) library and the Wiley library (Wiley-VCH Verlag GmbH & Co. KGaA, Weinheim, Germany). One feature ion was selected as the quant mass for acquiring the peak area for each compound, and the quantitative data were normalized by the IS. To identify the differences in the metabolic profiles between groups, the PLS-DA model and the OPLS-DA model were applied using SIMCA 13.0 (Umetrics, Umea, Sweden). In addition, Student’s *t*-test was applied to measure the significance of each compound. The open database source, MetaboAnalyst, was used to identify the metabolic pathways.

### Determination of Crocin and Crocetin in Biological Samples

The concentration of crocin and crocetin in biological samples was determined as previously described (Zhang et al., 2017). Briefly, 50 μL of plasma samples (or brain homogenate) was added to 5 μL of digoxin (2.0 μg/mL) and subsequently extracted by 1.0 mL of water-saturated *n*-butanol; then, the mixtures were vortex-extracted and centrifuged. The supernatant, 800 μL, was dried in a rotary evaporator and reconstituted with 100 μL of acetonitrile-water (v/v = 6:4), and an aliquot of 5 μL of supernatant was injected into the LC-MS/MS 8050 (Shimadzu, Japan) system for analysis. The chromatographic separation was achieved on a C18 column (100 mm × 2.1 mm, 3 μm, Atlantis^®^ T3, Waters). The mobile phase comprising 0.1% formic-acid solution (A) and acetonitrile (B) was delivered at a flow rate of 0.2 ml min-1 using a gradient program set as: initial 10% B for 1.0 min, linear gradient 10–65% B from 1.0 to 2.0 min, maintained 65% B for 2.5 min, linear gradient 65–75% B from 4.5 to 6.0 min, and to 95% B until 6.3 min, maintained 95% B until 8.0 min, and then returned back to 40% B from 8.0 to 8.8 min, maintained 40% B until 10.5 min, and then returned back to initial 10% B in 0.5 min and maintained for a further 2.0 min for column balance. Multiple reaction monitoring (MRM) was performed in the negative mode for the determination of crocin at m/z 975.45→651.20, crocetin at m/z 327.40→239.30, and the IS digoxin at m/z 779.50→649.40.

### Pharmacokinetic Studies

#### *In vitro* Metabolic System of Gut Flora

The anaerobic, *in vitro* metabolic system of gut flora was established and validated in a previous report (Zhao et al., 2014). The intact cecum content was removed and stored in an aqueous solution of 20% glycerol and 1.8% sodium chloride (1 g/3 mL). The procedure was sterile to avoid microbial contamination. The gut content solution was diluted 10-fold by Poly Peptone Yeast Extract Medium (PY culture medium). Tubes were filled by a gentle flow of N_2_, and subsequently incubated at 37°C for 12 h to allow the proliferation of the gut flora. For the *in vitro* metabolic analyses, 5 μL of crocin or crocetin (20 μM) were added to 45 μL of fermented gut content and incubated in an anaerobe culturing device (AnareoPack 2.5 L) for 0, 0.25, 0.5, 1, 1.5, and 2 h. After incubation, the samples were extracted and determined as described in Section “Determination of Crocin and Crocetin in Biological Samples.”

#### Excretion Study

Rats were administered either crocin (1 mg/kg, i.v.) or crocetin (0.33 mg/kg, i.v.), six rats in each group (three male, three female), and subsequently raised in metabolic cage separately with free access to food and water. The total urine and feces samples were collected every 8 h (i.e., 0–8, 8–16, 16–24, 24–32, 32–40, 40–48 h) after drug administration. The urine samples were collected directly while the feces samples were collected in 20 mL of 70% methanol, based on the stability validation (unpublished data).

The total volume of urine samples collected in each interval was measured, and 40 μL of urine was diluted 5-fold with 160 μL of distilled, deionized water. The diluted urine samples were subsequently extracted and determined as described in Section “Identification of Differential Metabolites Co-metabolic by Microbes and Host.” The total weight of the feces samples collected in each interval was measured and homogenized by supplying the required volume of distilled, deionized water (1:10, g/mL). The feces homogenate was diluted 50-fold with distilled, deionized water and subsequently extracted and determined as described in Section “Determination of Crocin and Crocetin in Biological Samples.”

#### Pharmacokinetic Studies in Control and pGF Rats

Rats received a cocktail of antibiotics for 6 days. The drugs (crocin, 58.6 mg/kg, and crocetin, 19.7 mg/kg) were again orally administered 24 h after final antibiotic administration to these pGF rats. For control rats, the same volume of 0.5% CMC-Na was given instead of antibiotics. Blood samples collection and preparation were performed as previously described (Zhang et al., 2017). Phoenix WinNonlin 6.3 pharmacokinetic program (Pharsight, Mountain View, CA, United States) was used to calculate the pharmacokinetic parameters.

### Identification of Differential Metabolites Co-metabolic by Microbes and Host

A total of 24 male rats were randomly divided into four groups, including control group, control with crocin (i.g., 58.6 mg/kg) group, pGF group, and pGF with crocin (i.g., 58.6 mg/kg) group. To minimize the impact of food intake, rats were fasted more than 12 h before crocin administration. After 6 h of crocin administration, the gut contents were collected and stored at -70°C until additional extraction and analysis.

For GC-MS metabolomic analysis, 150 mg gut content was mixed with ultrapure water 200 μL, vortexed for 5 min, and the mixture was centrifuged at 10,000 rpm for 10 min. Fifty μL of the supernatant was transferred to a new tube and added with 200 μL methanol (containing IS [^13^C_2_]-myristic acid, 5 μg/mL), and subsequently extracted and determined as described in Section “Metabolomics Study and Multivariate Statistical Analysis.”

The concentrations of SCFAs in gut content were relative quantified using an established GC-MS assay in our laboratory. The details of samples extraction and analytical method are provided in supporting materials.

Relative quantitation of available bile acids in gut contents was determined with a well-established and validated UFLC-Triple-TOF/MS assay in our laboratory (Zhou et al., 2014; Cao et al., 2017). Briefly, 150 mg of gut content was ultrasonic extracted in 500 μL of 70% ethanol for 4 h at 55°C. After centrifuging at 4,000 *g* for 10 min, 100 μL of the supernatant was added with 500 μL 0.1‰ formic acid (containing IS d_4_-cholic acid, 0.5 μg/mL), purified with Oasis-HLB cartridges, the eluate was evaporated and reconstituted in 100 μL methanol.

### Statistical Analysis

The data are expressed as the mean ± SD. Unpaired *t*-test was used to determine the significance of differences between two groups. The differences among multiple groups were evaluated using one-way analysis of variance (ANOVA). Bonferroni was used as a *post hoc* test when appropriate for one-way ANOVAs and statistical significance was set at *P* < 0.05 (GraphPad Prism 7.0).

## Results

### Oral Administration of Crocin Elicits Superior Cerebral-Protective Effects Over Intravenous Administration

**FIGURE 2:**
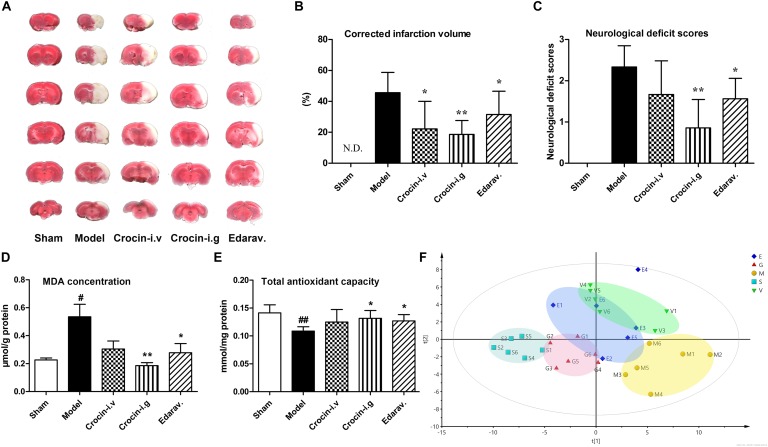
Oral administration of crocin elicits superior cerebral-protective effects over intravenous administration. **(A)** Representative images of six serial sections from the same brain (top to bottom) prepared 24 h after MCAO and stained with TTC. The red areas represent normal brain tissues, and the white areas represent the infarct tissues. **(B)** Average infarction volumes corrected for brain edema as indicated (*n* = 6), N.D., infarction not detected. ^∗^*P* < 0.05, ^∗∗^*P* < 0.01 vs. model group. **(C)** Neurological deficit scores in each group. ^∗^*P* < 0.05, ^∗∗^*P* < 0.01 vs. model group. **(D)** Malondialdehyde (MDA) and **(E)** total antioxidant capacity of brain tissues in the infarction region. Data are presented as the mean ± SD (*n* = 6), ^#^*P* < 0.05, ^##^*P* < 0.01 vs. sham group; ^∗^*P* < 0.05, ^∗∗^*P* < 0.01 vs. model group. **(F)** PLS-DA scores plots of brain tissues collected 24 h after MCAO. S, sham-operated group; M, model group; E, edaravone; G, crocin (i.g. administration) group; V, crocin (i.v. administration) group.

The traditional approach for saffron intake is oral administration, but the bioavailability of orally administered crocin, the main water-soluble component, is only 0.3‰∼0.6‰. Thus, two ways of drug delivery, oral and intravenous administration, were adopted to evaluate the protective effect of crocin on cerebral I/R injury. In addition, edaravone, a free radical scavenger and well-tested neuroprotective compound, was selected as a positive control. Representative TTC-stained coronal brain sections from all experimental groups are shown in Figure 2A, and the statistics after correcting for brain edema are shown in Figure 2B. Compared to the model group, oral administration of crocin showed a significant reduction in the cerebral infarction volumes (*P* = 0.001). Edaravone was also completely effective at reducing cerebral infarction (*P* = 0.028), while intravenous administration of crocin showed less effective to reduce brain infarction volumes with great individual differences. Moreover, crocin (i.g. administration) and edaravone treatment produced a trend toward neurological behavior improvements compared to the sham-operated group. In addition, inferior improvement was observed after i.v. administration of crocin, Figure 2C.

Both crocin and edaravone possess unique antioxidant capacity, and hence the MDA level and total antioxidant activity in the damaged brain region were investigated. As indicated in Figure 2D, the content of brain MDA increased markedly in the model group, compared with the sham-operated group. Crocin (i.g. administration) treatment significantly attenuated the increase in MDA level, as did the edaravone treatment. The intravenous administration of crocin could also reduce the MDA level but without significant difference. Similarly, the total antioxidant activity was significantly reduced in the model group, while crocin (i.g. administration) and edaravone treatment markedly increased the total antioxidant activity relative to the sham-operated group (Figure 2E). In addition, there was no significant improvement in the crocin (i.v. administration) group.

We further investigated the differences of metabolite profile in the injure brain region among groups. A total of 82 endogenous metabolites were identified in the brain tissue samples. The PLS-DA model was applied to characterize the metabolic disturbances among groups (Figure 2F). The quantitative parameters were obtained for the following: R^2^X at 0.85, R^2^Y at 0.976, and Q^2^ at 0.489, indicating a good fitness and prediction. As illustrated in Figure 2F, the close clustering within each group represents higher compositional similarity. Whereas the great distance between the sham-operated group and model group represents diverse metabolomic compositions, indicating that cerebral I/R injury markedly disturbed the metabolism of brain. The crocin (i.g. administration) group is completely separated from the model group and closer to the sham-operated group, implying that the i.g. administration of crocin distinctly ameliorated the dysfunctional metabolism of the brain. Both edaravone and crocin (i.v. administration) treatment were less effective than crocin (i.g. administration) treatment at intervening in the disturbed metabolism of brain.

Clear differences were obtained from the OPLS-DA model when we focused on the model group vs. the sham-operated group for cerebral I/R injury; and the crocin (i.g. administration) group vs. the model group for the crocin treatment. The metabolites with *P*-values < 0.05 were considered the potentially differential metabolites. Detailed information about the potentially differential metabolites, such as compound name, retention time, mass-to-charge ratio, VIP value, fold change, and *P*-value for each comparison appear in Supplementary Table S1. Pathway analysis and enrichment analysis were obtained from these potential differential metabolites with MetaboAnalyst 3.0^1^. For the model group vs. the sham-operated group, the enrichment analysis (Supplementary Figure S1A) and pathway analysis (Supplementary Figure S1B), metabolism changed for protein biosynthesis, amino acids metabolism, glycolysis, and citrate cycle. Similar metabolic pathways were obtained for the crocin (i.g. administration) group vs. the model group (Supplementary Figures S1C,D), indicating the modulation of brain metabolites to normal levels by crocin (i.g. administration) treatment. Specifically, the disturbance of amino acid metabolism was the most significant in both paired comparisons. Most amino acids, especially the branched chain amino acids, were significantly increased in the model group, indicating that hypoxia led to the damage of enzyme functions and activities involved in branched chain amino acid metabolism in the brain. And most of these amino acids were rectified by crocin (i.g. administration) treatment.

### Neither Crocin nor Its Metabolite, Crocetin, Could Enter the Brain in the Normal and Cerebral I/R Rats

As shown in the previous sections, our data clearly shows that crocin (i.g. administration) treatment has better protective effects than edaravone on cerebral I/R injury and is obviously superior to crocin (i.v. administration) treatment. However, previous pharmacokinetic studies demonstrated that crocin is hard to be absorbed in circulation after oral administration. But crocin can be transformed into crocetin in the gastrointestinal tract, and the plasma exposure of crocetin is 56- to 81-fold higher than crocin. Meanwhile, no crocetin was determined in plasma after intravenous administration of crocin (Zhang et al., 2017; Yu et al., 2018). Which raises this question: Whether the cerebral I/R injury has a functional impact on the absorption property of crocin, which leads to more metabolite (crocetin) in blood circulation, and also impact the ability of crocin and crocetin to across the blood-brain barrier?

**FIGURE 3:**
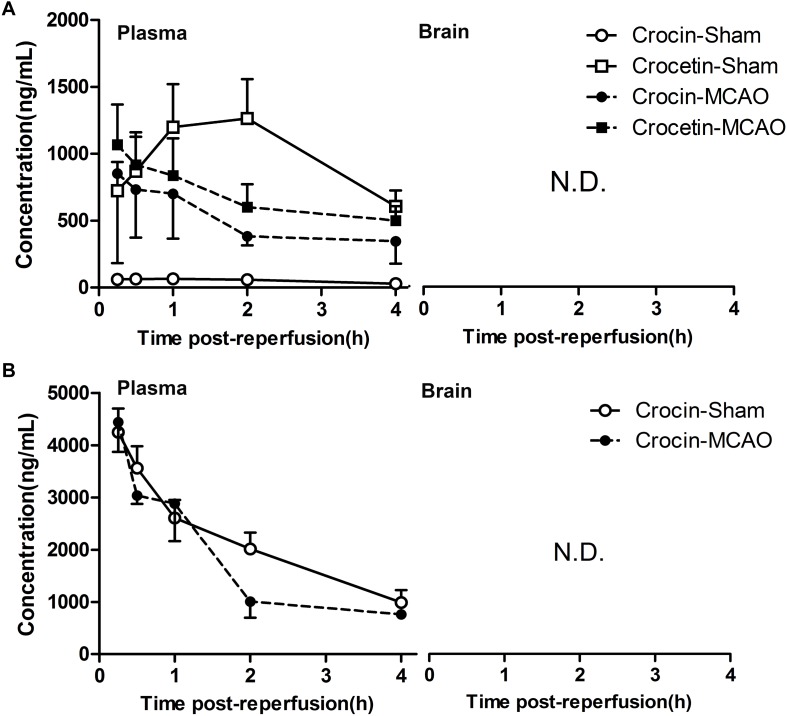
Neither crocin nor its metabolite, crocetin, could enter the brain in the normal and cerebral I/R rats. **(A)** Oral administration of crocin (60 mg/kg). **(B)** Intravenous administration of crocin (1 mg/kg). Data are expressed as the mean ± SD (*n* = 5). N.D., under the lower limit of detectability (2 ng/mL).

To investigate this question, we determined the cerebral and plasma exposure of crocin and crocetin in normal and cerebral I/R rats. As a result, after oral administration of crocin, a higher plasma concentration of crocin and a lower plasma concentration of crocetin were observed in cerebral I/R rats, as compared with the normal group (Figure 3A). However, the plasma concentration of crocin was still far below that of the intravenous administration (Figure 3B). Additionally, either way, no crocin or crocetin was detected in the cerebral tissue. These results revealed that crocin will not take effect in the cerebral tissue through its prototype or metabolite, and most importantly, the crocetin in the circulatory system may account for the cerebral-protective effects of crocin.

### No Significant Protective Effects of Intravenously Administered Crocetin on Cerebral I/R Injury

**FIGURE 4:**
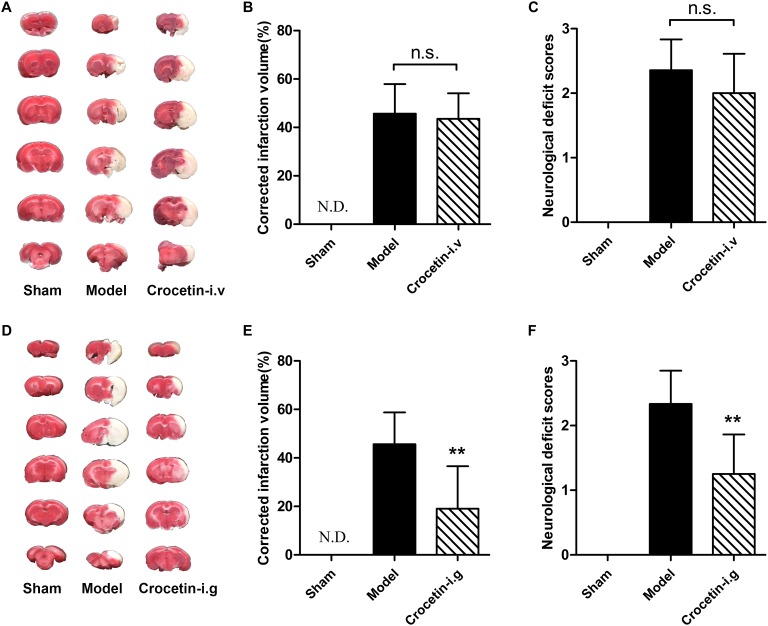
The effect of crocetin on cerebral I/R injury. Crocetin was treated at equal molar dose to crocin (0.33 mg/kg for intravenous administration and 20 mg/kg for oral administration). Representative images of the same brain stained with TTC **(A)**, average infarction volumes corrected for brain edema as indicated **(B)** and the neurological deficit scores in each group **(C)** are shown after the intravenous administration of crocetin. Data are represented as the mean ± SD (*n* = 6), N.D., infarction not detected. Representative images of the same brain stained with TTC **(D)**, average infarction volumes corrected for brain edema as indicated **(E)** and neurological deficit scores for each group **(F)** are shown after the oral administration of crocetin. Data are presented as the mean ± SD (*n* = 6), N.D., infarction not detected. ^∗∗^*P* < 0.01 vs. model group.

We next hypothesized that the cerebral-protective effect of the oral administration of crocin is due to the exposure of crocetin in circulation. To test this hypothesis, we investigated the effect of intravenous crocetin administration on cerebral I/R injury. It turned out that direct intravenous administration of crocetin could not reduce brain infarction volume or improve neurological behavior (Figure 4A–C). However, similar to crocin, oral administration of crocetin produced significant cerebral-protective effects (Figure 4D–F). Together, these lines of evidence suggest that the target/signaling pathway in the gut can be modulated by crocin and that crocetin is associated with crocin’s protective effect against cerebral I/R injury.

### Intravenously Administered Crocin and Crocetin Were Not Excreted Through the Gut

To further elucidate whether the target of the cerebral-protective effect of crocin is in the gut, we addressed if the intravenous administration of crocin or crocetin is eliminated without excretion into the gut. As a result, after the intravenous administration, crocin was mainly excreted in the urine, with a cumulative excretion fraction of 67.17 ± 4.79% within 48 h (Supplementary Figure S2A). And no crocin was detected in the fecal samples (Supplementary Figure S2B). Crocetin was not excreted in the urine or feces after intravenous administration (Supplementary Figures S2C,D). Furthermore, orally administered crocin was mainly excreted in the feces in the form of its metabolite crocetin, and crocetin was also mainly excreted in the feces after oral administration (unpublished data). These results were in accordance with the significant cerebral-protective effect of crocin and crocetin through oral administration and inferiority of intravenous administration, suggesting that the potential target of the cerebral-protective effect of orally administered crocin and crocetin is in the gut.

### Less Cerebral-Protective Effect of Crocin on Cerebral I/R Injury in pGF Rats

**FIGURE 5:**
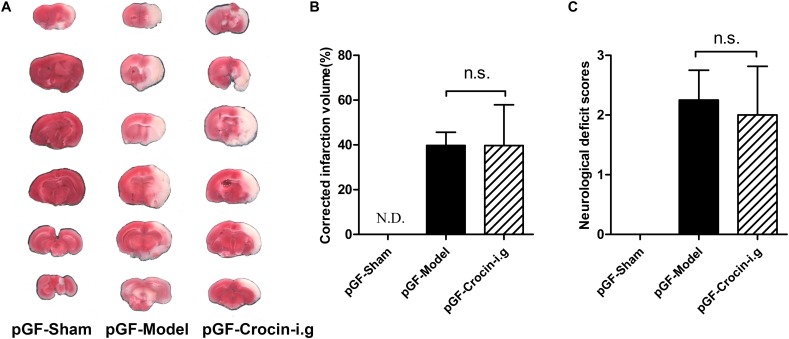
The effect of orally administered crocin on cerebral I/R injury in pGF rats. Representative images of the same brain stained with TTC **(A)**, average infarction volumes corrected for brain edema as indicated **(B)**, and neurological deficit scores in each group **(C)** are shown after orally administered crocin in pGF rats. Data are represented as the mean ± SD (*n* = 6), N.D., infarction not detected.

The emerging pivotal role of the gut flora in the pathogenesis of ischemic stroke strongly prompts the speculation that the gut flora could be a therapeutic target. pGF rats, achieved by broad-spectrum antibiotics treatment to largely eradicate the gut flora, were widely adopted in elucidating the role of gut flora in host pathophysiology and the metabolism of xenobiotics. Then, the effect of crocin on cerebral I/R injury was delineated in pGF rats. It turned out that no beneficial effect of orally administered crocin on reducing brain infarction volume or improving neurological behavior (Figure 5A–C) was observed in pGF rats.

### Metabolism of Crocin and Crocetin by Gut Flora *in vitro*

**FIGURE 6:**
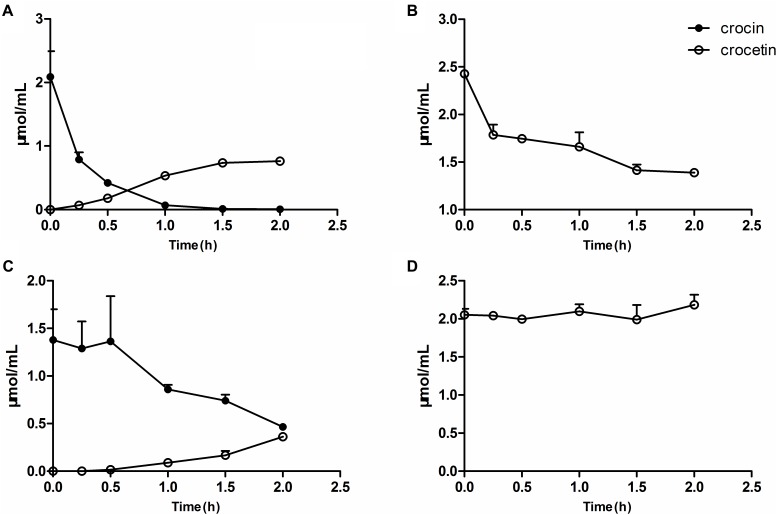
Metabolism of crocin and crocetin by gut flora *in vitro*. The metabolism of crocin **(A)** and crocetin **(B)** by the gut flora from normal rats. The metabolism of crocin **(C)** and crocetin **(D)** by the gut flora from pGF rats. Data are represented as the mean ± SD (*n* = 5).

The measurement of the metabolism of crocin *in vitro* revealed that crocin could be easily deglycosylated to crocetin by the gut flora, meanwhile, crocetin was also metabolized by the gut flora. The residual amount remaining after interaction with gut flora for 2 h (Figure 6A,B) was in accordance with the *in vivo* excretion results showing that orally administered crocin is mainly excreted in the form of crocetin (unpublished data), suggesting that the *in vitro* metabolic system has a good ability to predict the metabolic activity of gut flora after oral administration *in vivo*. Herein, the *in vitro* metabolic system was employed to elucidate the metabolic status of crocin and crocetin in the intestines of pGF rats. As shown in Figure 6C,D, trace levels of crocetin could be found after the incubation of crocin with the gut flora of pGF rats. Meanwhile, the gut flora of pGF rats was no longer capable of metabolizing crocetin. After the oral administration of crocin, due to its poor absorption by enterocytes, a considerable portion of crocin was stranded in the gut where it can be directly metabolized into crocetin by the gut flora. The interaction of crocetin with the gut flora suggests to be the mechanistic understanding of the systemic effects of crocin. It was suggested that the gut flora plays a key role in the transformation of crocin into crocetin and is the potential target for the cerebral-protection of crocin in MCAO model rats.

### The Cross-Interaction Between Gut Microbiota and Crocin Was Abolished in pGF Rats

**FIGURE 7:**
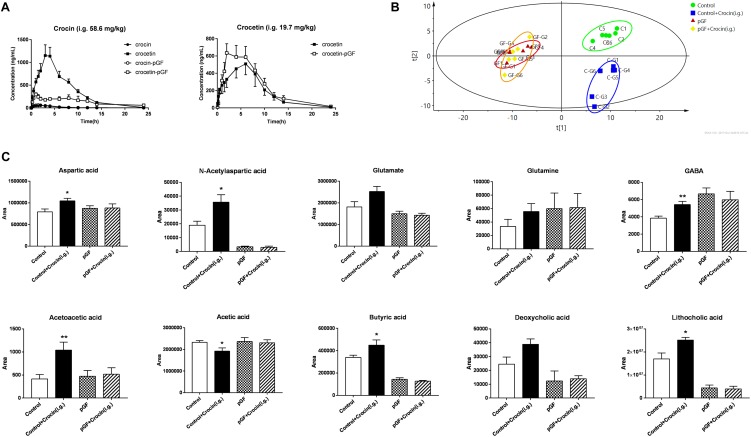
The cross-interaction between gut microbiota and crocin was abolished in pGF rats. **(A)** PK curves of crocin and crocetin after oral administration (crocin, 58.6 mg/kg; crocetin, 19.7 mg/kg) (mean ± SD, *n* = 6); **(B)** PLS-DA scores plots of gut content collected 6 h after crocin or vehicle administration. **(C)** The metabolites identified to be significantly different between crocin administered and control rats, (mean ± SD, *n* = 6). ^∗^*P* < 0.05, ^∗∗^*P* < 0.01 vs. control group.

To further investigate the relationship between gut microbiota and crocin, the pharmacokinetic studies of crocin and crocetin in pGF rats were compared with control rats. Unsurprisingly, significant lower concentration of crocetin in systemic circulation was determined after oral administration of crocin in pGF rats, compared with that of control rats, indicating that much less crocin was deglycosylated to crocetin by gut microbiota of pGF rats, Figure 7A. No significant difference of the pharmacokinetic properties of crocetin was found between control rats and pGF rats. The main pharmacokinetic parameters were calculated and showed in Supplementary Tables S2, S3. A PLS-DA model was applied to obtain the classification performance with a total of 140 bacterial-host co-metabolic products, Figure 7B, and the model parameters were R^2^X = 0.752, R^2^Y = 0.998, *Q*^2^ = 0.708, indicating a good fitness and prediction. The control with crocin administration group is apparently separated from the control group, while the pGF with crocin administration group is indistinguishable from the pGF group. Some metabolites involved in energy metabolism, glutamine-glutamate-GABA metabolic pathway, and anti-inflammatory response were identified to be significantly different between crocin administered and control rats, while no change was found in pGF rats with and without crocin administration, Figure 7C.

## Discussion

**FIGURE 8:**
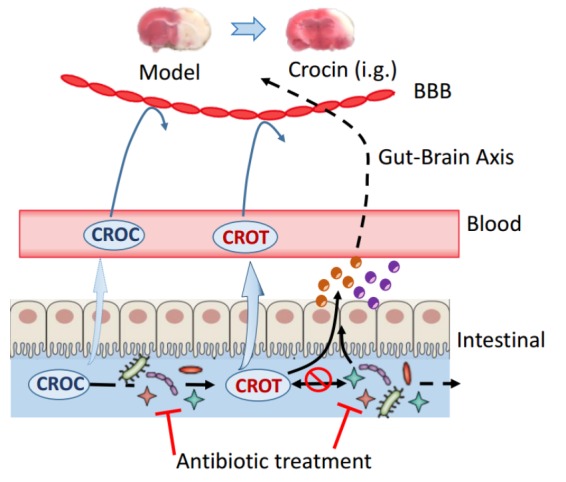
Proposed active target of orally administered crocin on cerebral I/R injury. Oral administration of crocin elicits a superior cerebral protective effect over intravenous administration, and both crocin and crocetin could not cross the blood brain barrier even in rats with cerebral I/R injury. Direct intravenous administration of crocetin could not reduce the brain infarction volume or improve neurological behavior indicating that the crocetin in circulation system does not account for the cerebral-protective effects of crocin. Crocin could be easily deglycosylated to crocetin by gut flora, meanwhile, crocetin was also metabolized by the gut flora. However, trace levels of crocetin could be found after the incubation of crocin with the gut flora of pGF rats, and the gut flora of pGF rats was no longer capable to metabolize crocetin. Additionally, the cerebral-protective effect of orally administered crocin disappeared in pGF rats. Our study from the insight of reverse pharmacokinetics hints to the possibility that the gut flora might be the potential target of crocin and crocetin, although the details connecting the gut flora and stroke prognosis remain largely unclear. BBB, blood brain barrier; CROC, crocin; CROT, crocetin; Brown and purple circles represent some bioactive components.

Pharmacokinetic and pharmacodynamic studies revealed that crocin and crocetin in the gut played a key role in the activation of the pharmacological effect. *In vitro* and *in vivo* studies conformed that remarkable lower level of crocin was transformed into crocetin by the gut microbiota of pGF rats, accompanied by abolished cerebral-protective effect in pGF rats. In light of these evidences, we proposed that gut microbiota plays a vital role in the disposition of crocin and crocetin in the gastrointestinal tract (Figure 8) and strongly associates with the cerebral-protective effect of crocin. In addition, some endogenous metabolites were significantly perturbed by crocin administration of in control rats, which are potentially involved in the mechanism for the cerebral-protective effect of crocin and crocetin.

Accumulating evidence reveals a close linkage between gut microbiota and stroke outcome, and the contribution of the gut microbiota to brain function has gained considerable interest (Winek et al., 2016b,c). A line of experimental evidence has shown that stroke markedly affects the gut microbiota’s composition, such as a reduction species and overgrowth of Bacteroidetes (Singh et al., 2016), and the integrity of the intestinal mucosal barrier (Stanley et al., 2016). Subsequently, the disturbed gut microbiota induces peripheral immune activation, a substantial induction of proinflammatory Th1 and Th17 T_helper_ cell polarization, which can be trafficked in circulation (Benakis et al., 2016). As a result, dysbiosis of the gut microbiota in turn affects stroke outcome via immune-mediated mechanisms (Winek et al., 2016a). While, bacteria rely heavily on small molecules to interact with the host (Meinwald and Eisner, 2008). Recently, several exact signaling molecules produced in the gut have been found which mediate the effect of gut microbiota on the host health. *N*-acyl amides, the metabolites of bacterial, can interact with GPCRs and subsequently regulate metabolic hormones and glucose homeostasis of the host (Cohen et al., 2017). Besides, desaminotyrosine (DAT), produced by a specific gut microbe from the digestion of plant flavonoids, protects the host against influenza through modulation of type I IFN (Steed et al., 2017).

In this study, the metabolites produced by the host and bacteria were significantly perturbed after oral administration of crocin, which may have strong system effect (Aoki-Yoshida et al., 2016; Khorasany and Hosseinzadeh, 2016; Menghini et al., 2018). Aspartate was reported to protect the enterocytes against oxidative stress (Turut et al., 2009; Sivakumar et al., 2011). *N*-acetylaspartate is a biomarker that directly reflect the degree of neuronal injury in cerebral (Igarashi et al., 2015), however, whether the increased level of *N*-acetylaspartate in the gut is directly associated with the level in cerebral needs further study. Glutamine is the most important oxidative fuel of enterocytes which can promote the regeneration of mucosal cells and improve the function of intestinal mucosal barrier. Glutamate, functions as a key enteric neurotransmitter, may have therapeutic potential for improving gut function (Burrin and Stoll, 2009). The concentration of GABA was significantly increased after crocin treatment, and the effects of GABA receptors are strongly involved in brain ischemic injury and repair, but the mechanism is complicated which need further investigation. Additionally, both SCFAs and secondary bile acids produced by gut microbiota in the gut mediate the anti-inflammatory response in periphery. Butyric acid was found to facilitate the generation of anti-inflammatory Treg cells (Arpaia et al., 2013) and accumulated secondary bile acid, such as deoxycholic acid and lithocholic acid, could activate TGR5 (also known as GPBAR1) and trigger anti-inflammatory response through cAMP-PKA pathway (Cipriani et al., 2011; Biagioli et al., 2017).

The brain metabolomic analysis revealed there was a sharp distinction of the metabolite profiles of the brain between the cerebral I/R rats and sham-operated rats. Furthermore, i.g. administration of crocin distinctly ameliorated the dysfunctional metabolism of the brain to normal status. The serum metabolomic analysis data were not shown since the metabolite profile in the serum of the cerebral I/R rats at 24 h after I/R was very close to that of the sham-operated rats (Geng et al., 2017). It remains to be elucidated that the sample pGF rat model can provide a temporary, almost completely microbe-free intestinal environment, however, there are significant challenges in the maintenance of the germ-free environment (Sousa et al., 2008; Kang et al., 2013; McCabe et al., 2015). Germ free rats should be chosen to further verify both the role of the gut microbiota in the metabolism of crocin and crocetin and of the bidirectional microbiota–crocetin interaction (Wilson and Nicholson, 2017).

There was a holy grail that all drugs should have an appropriate exposure level and time surrounding their target organs, however, the molecular targets for most natural medicines are elusive (Smoliga et al., 2012; Hao et al., 2014). Although the underlying mechanisms of the potential active metabolites in the gut are not understand yet, our data add to the connection that gut microbiota play a vital role in the activation of the pharmacological effect of crocin. Our study can be a very good template for subsequent natural medicines with therapeutic benefits but possess undesirable pharmacokinetic profiles. Thus avoiding to use impractical high concentrations to address the target/mechanism with *in vitro* studies, which have poor clinical translational potentials.

## Conclusion

Collectively, pharmacokinetic and pharmacodynamic association studies provide evidence that the gut microbiota plays a vital role in the fate of crocin and crocetin in the gastrointestinal tract. In addition, the cross-interaction between gut microbiota and crocin might mediate the activation of the cerebral-protective effect of orally administered crocin.

## Ethics Statement

This study was carried out in accordance with the recommendations of ‘Institutional Animal Research Committee guidelines, Animal Ethics Committee of China Pharmaceutical University.’ The protocol was approved by the ‘Animal Ethics Committee of China Pharmaceutical University.’

## Author Contributions

JA, GW, and YZ participated in research design. YZ, YH, JG, LJ, SL, and CY conducted the experiments. YZ, JG, RS, and YX performed the data analysis. YZ, JA, and GW wrote or contributed to the writing of the manuscript. All authors read and approved the final manuscript.

## Conflict of Interest Statement

The authors declare that the research was conducted in the absence of any commercial or financial relationships that could be construed as a potential conflict of interest.

## Footnotes

1 http://www.metaboanalyst.ca/faces/ModuleView.xhtml

## Abbreviations

BBB: blood brain barrier
CROC: crocin
CROT: crocetin
ES: external standard
GABA: γ-aminobutyric acid
GC-MS: gas chromatography-mass spectrometer
i.g.: intragastric
i.p.: intraperitoneal
i.v.: intravenous
I/R: ischemic/reperfusion
IS: internal standard
MCAO: middle cerebral artery occlusion
MDA: malondialdehyde
OPLS-DA: orthogonal projection to latent structure-discriminant analysis
pGF: pseudo germ-free
PLS-DA: partial least squares-discriminant analysis
rt-PA: recombinant tissue-type plasminogen activator
SCFA: short chain fatty acid
