# Supplementary material for: Orally Administered Crocin Protects Against Cerebral Ischemia/Reperfusion Injury Through the Metabolic Transformation of Crocetin by Gut Microbiota
Source: Front Pharmacol. 2019 Apr 30;10:440. doi: 10.3389/fphar.2019.00440 (PMC6502977; doi:10.3389/fphar.2019.00440)
Supplement: Supplementary file 1 [file Table_1.PDF]

Supplementary Information

Relative quantitation of SCFAs in gut contents

The GC-MS assay for determination of 11 SCFAs in gut content was applied for a Chinese patent which was published on June 8, 2016 by Hao HP, *et al,* "Simultaneous assessment of 11 SCFAs in gut content and fece samples by GC-MS". In detail, approximately 150 mg of gut contents was acidized by 100 μL 1 M HCL which was prepared with saturated sodium chloride solution. Then 1 mL ethyl acetate (containing IS 2-ethyl butyrate, 5 μg/mL) was added and vortexed for 5 min. The mixture was centrifuged at 18,000 rpm for 10 min and 220 μL of the supernatant was dried with 100 mg anhydrous $MgSO_4$. Then 150μL of the supernatant was dried again with 50 mg anhydrous $MgSO_4$. After centrifugation at 18000 rpm for 10 min, 70 μL of the supernatant was transferred into the GC autosampler vials and added with 18 μL N-tert-butyldimethylsilyl-N-methyltrifluoracetamide (MTBSTFA). Then the mixture was incubated for 20 min at 80 ℃, followed by 8 hours at room temperature for derivatization and was then ready for GC-MS analysis.

The derivatized samples were analyzed using gas chromatography coupled to a mass spectrometer (Shimadzu GCMS-QP2010 Ultra, Kyoto, Japan) equipped with an automatic sample injector (Shimadzu AOC-20i, Kyoto, Japan). A 0.5-μL sample aliquot was injected into the Rtx-5MS capillary column (0.25 mm × 30 m × 0.25 μm, Restek, PA, USA) in splitless mode. The injector temperature was set at 270 ℃. The septum purge was turned on with a flow rate of 2.0 mL/min. Helium was used as the carrier gas, with a flow rate of 1.0 mL/min. The column temperature was initially maintained at 40 °C for 3 min, then raised to 60 °C at a rate of 40 °C/min, and then raised to 110 °C at a rate of 10 °C/min, and then raised to 250 °C at a rate of 40 °C/min, and held for 1 min. Each run was a total of 14 min, and two solvent cutting acquisition were set at 0-4.5 min and 7.0-7.6 min. The mass spectrometer ion source temperature was 250 °C, and ionization was achieved with a 70 eV electron beam. Mass spectra were obtained in a SIM mode. The qualitative ion and retention time for the SCFAs were as follows: formic acid, m/z 46, 5.125 min; acetic acid, m/z 60, 6.240 min; propionic acid, m/z 74, 7.690 min; butyric acid, m/z 71, 8.850 min; isobutyric acid, m/z 71, 8.22 min; valeric acid, m/z 85, 9.730 min; isovaleric acid, m/z 87, 9.35 min; hexanoic acid, m/z 116, 10.425 min; isohexanoic acid, m/z 116, 10.135 min; lactic acid, m/z 59, 11.425 min; succinic acid, m/z 55, 12.150 min; 2-ethylbutyric acid (IS), m/z 116, 9.920 min.

Table S1 The detailed information about the potential differential metabolites.

Differential metabolites	Retention time (min)	Mass-to-charge ratio	VIP	Fold change	*P* value
**Model vs. Sham**					
Lactic acid	4.985	191	3.589	1.680	<0.001
N-Acetylaspartic acid	9.685	158	2.929	0.648	0.020
Alanine	5.460	116	2.490	1.559	0.015
Serine	7.755	204	2.437	1.577	0.001
Glutamine	10.355	156	2.322	1.609	0.047
Urea	6.730	189	2.046	1.380	0.080
Leucine	7.050	158	1.798	2.197	0.003
Glucose-6-phosphate	13.130	387	1.629	12.889	0.008
Proline	7.275	142	1.584	2.177	0.007
Tyrosine	11.255	218	1.447	1.711	0.008
Pyroglutamic acid	8.890	156	1.368	1.079	0.161
Isoleucine	7.235	158	1.341	2.168	<0.001
Glycine	7.350	174	1.299	1.089	0.120
Phenylalanine	9.545	218	1.274	1.787	0.003
Fructose-6-phosphate	13.080	315	1.272	7.510	0.007
Glycerol-2-phosphate	10.455	299	1.211	0.780	0.026
L-Lysine	11.155	317	1.130	1.930	0.002
Threonine	7.960	218	1.115	1.481	0.018
Fructose	10.975	307	0.947	8.340	<0.001
Tryptophan	12.635	202	0.924	2.612	0.012
Palmitic acid	11.690	313	0.877	1.121	0.014
Aspartic acid	8.850	232	0.876	0.937	0.430
Cholesterol	17.535	458	0.786	0.904	0.024
D-Glucopyranose-6-Phosphate	13.225	387	0.777	3.936	0.011
Glucose	11.115	319	0.742	6.278	0.008
Pyruvic acid	4.845	174	0.703	0.440	0.001
Glutamic acid	9.460	246	0.702	1.037	0.390
Histidine	11.175	254	0.677	4.391	0.015
Malic acid	8.640	233	0.646	1.255	0.050
Niacinamide	8.575	179	0.631	0.767	0.048
Valine	6.580	218	0.593	1.975	<0.001
Glucitol	11.330	319	0.571	2.018	0.007
Cystathionine	12.545	218	0.564	4.006	0.013
Fumaric acid	7.595	245	0.538	0.859	0.088
2-Ketoglutaric acid	10.835	173	0.533	0.837	0.067
Octadecanoic acid	12.600	341	0.524	1.067	0.206
**i.g vs. Model**					

N-Acetylaspartic acid	9.685	158	3.547	1.509	0.012
Alanine	5.460	116	2.827	0.670	0.024
Urea	6.730	189	2.761	1.556	0.310
Serine	7.755	204	2.585	0.692	0.006
Leucine	7.050	158	1.926	0.568	0.004
Glycerol-2-phosphate	10.455	299	1.723	1.346	0.010
Proline	7.275	142	1.700	0.553	0.014
Tyrosine	11.255	218	1.660	0.626	0.009
Threonine	7.960	218	1.577	0.610	0.005
Isoleucine	7.235	158	1.434	0.575	<0.001
Inosine	14.085	281	1.378	1.530	<0.001
Phenylalanine	9.545	218	1.368	0.635	0.007
L-Lysine	11.155	317	1.340	0.546	0.002
Serine	7.755	204	1.313	0.692	0.006
Glutamic acid	9.460	246	1.264	1.053	0.199
Glycine	7.350	174	1.227	0.948	0.142
Cholesterol	17.535	458	1.116	1.123	0.004
Aspartic acid	8.850	232	1.035	1.063	0.427
Fumaric acid	7.595	245	0.935	1.245	0.012
2-Ketoglutaric acid	10.835	173	0.927	1.293	0.006
Lactic acid	4.985	191	0.871	0.951	0.361
Palmitic acid	11.690	313	0.850	0.918	0.052
Tryptophan	12.635	202	0.765	0.618	0.081
Fructose	10.975	307	0.719	0.526	0.026
Histidine	11.175	254	0.716	0.350	0.035
Gama-Aminobutyric acid	8.925	304	0.699	1.055	0.545
Pyroglutamic acid	8.890	156	0.632	0.976	0.550
Valine	6.580	218	0.625	0.626	<0.001
Uracil	7.58	241	0.617	0.861	0.108
Niacinamide	8.575	179	0.610	1.207	0.071
Glucose-6-phosphate	13.130	387	0.585	0.732	0.500
Succinate	7.360	247	0.582	0.562	0.014
Cystathionine	12.545	218	0.576	0.399	0.036
Phosphoric acid	7.080	314	0.554	0.630	0.027

Table S2

Pharmacokinetic parameters of crocin and its metabolite crocetin in rats after i.g. (58.6 mg/kg) administration of crocin (mean ± SD, n = 6)

**Parameters**		**control**		**pGF**	
		crocin	crocetin	crocin	crocetin
C_{max}	μg/L	56.0 ± 34.3	1278.1 ± 623.3	67.8 ± 28.2	303.2 ± 95.4**
T_{max}	h	2.6 ± 0.8	3.9 ± 1.0	1.6 ± 0.7*	1.1 ± 0.2***
$t_{1/2}$	h	2.9 ± 1.5	2.4 ± 0.5	3.5 ± 1.4	4.5 ± 1.5**
$AUC_{0\sim24h}$	μg/L/h	310.1 ± 212.2	8668.6 ± 2743.5	337.2 ± 81.2	3009.6 ± 2012.0**
$AUC_{0\sim\infty}$	μg/L/h	322.8 ± 216.6	8707.5 ± 2742.4	345.0 ± 81.2	3403.0 ± 2395.4**
MRT	h	4.4 ± 0.9	6.3 ± 0.9	5.4 ± 1.2	9.4 ± 3.0*
CL	L/h	246.0 ± 133.7	7.6 ± 3.2	176.3 ± 33.2	22.4 ± 9.6**
V	L	890.4 ± 404.3	26.9 ± 16.3	902.7 ± 441.4	135.0 ± 63.3***

Table S3

Pharmacokinetic parameters of crocetin in rats after i.g. (19.7 mg/kg) administration of crocetin (mean ± SD, n = 6)

**Parameters**		**control**	**pGF**
C_{max}	μg/L	566.7 ± 386.5	794.3 ± 252.2
T_{max}	h	5.4 ± 1.5	3.3 ± 2.1*
$t_{1/2}$	h	2.5 ± 0.8	2.7 ± 0.6
$AUC_{0\sim24h}$	μg/L/h	4586.9 ± 3009.4	6003.1 ± 2304.4
$AUC_{0\sim\infty}$	μg/L/h	4625.5 ± 3007.7	6055.6 ± 2314.5
MRT	h	3.4 ± 0.4	3.2 ± 0.2
CL	L/h	6.3 ± 4.5	3.9 ± 2.2
V	L	25.9 ± 27.8	15.4 ± 10.3

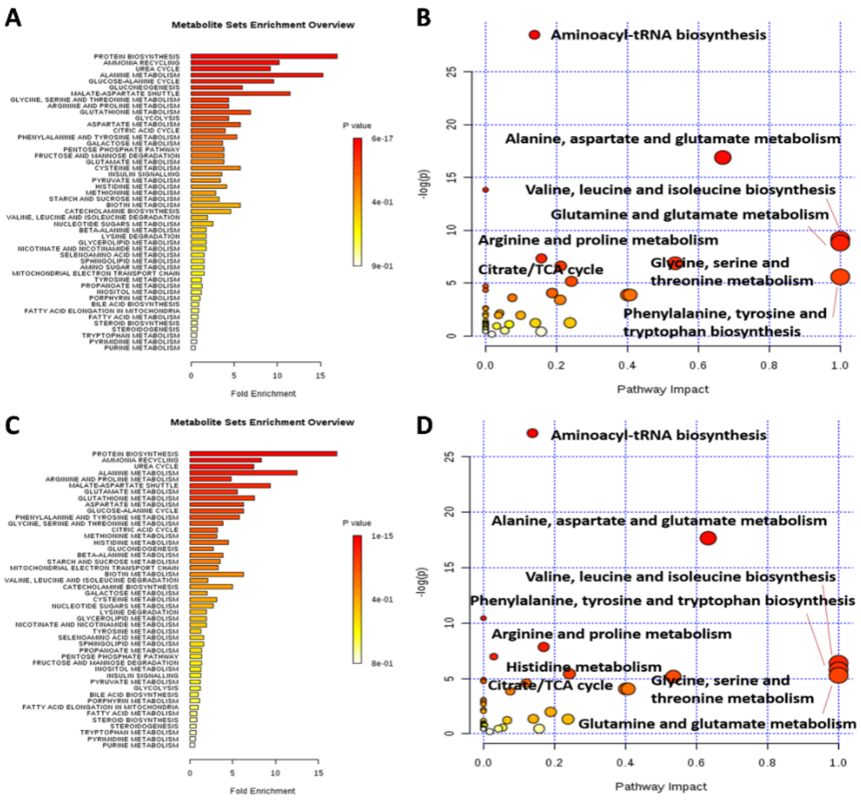

Fig. S1

Fig. S1 The impact analysis shows the effect of cerebral I/R injury and oral administration of crocin on primary metabolic pathway based on the metabolome of brain tissues. (A) Enrichment analysis of metabolic sets induced by cerebral I/R injury relative to sham-operated group. (B) Impact analysis of metabolic sets induced by cerebral I/R injury relative to sham-operated group. (C)Enrichment analysis of metabolic sets induced by oral administration of crocin relative to cerebral I/R injury group. (D) Impact analysis of metabolic sets induced by oral administration of crocin relative to cerebral I/R injury group.

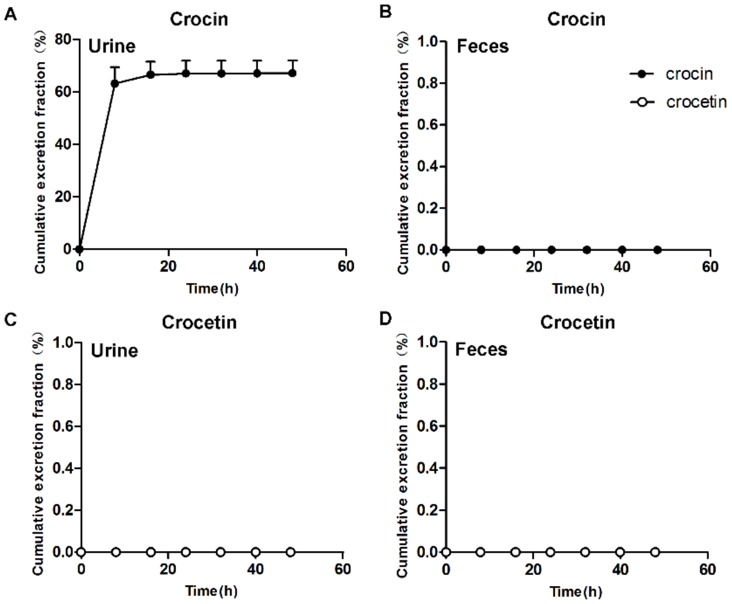

Fig. S2

Fig. S2 The excretion pathway of crocin and crocetin after intravenous administration. The cumulative excretion fraction of crocin in the urine (A) and feces (B). The cumulative excretion fraction of crocetin in the urine (C) and feces (D). Data are presented as the mean±SD (n=6).
